# Supplementary material for: Uncovering the rewired IAP-JAK regulatory axis as an immune-dependent vulnerability of LKB1-mutant lung cancer
Source: Nat Commun. 2025 Mar 8;16:2324. doi: 10.1038/s41467-025-57297-5 (PMC11890758; doi:10.1038/s41467-025-57297-5)
Supplement: Supplementary file 1 — Supplementary Information [file 41467_2025_57297_MOESM1_ESM.pdf]

Supplemental Information

Uncovering the rewired IAP-JAK regulatory axis as an immune-dependent vulnerability of LKB1-mutant lung cancer

Changfa Shu[1,2], Jianfeng Li[1], Rui Jin[3], Dacheng Fan[1], Qiankun Niu[1], Ruiyang Bai[1,4], Danielle Cicka[1], Sean Doyle[1], Alafate Wahafu[1,5], Xi Zheng[1,6], Yuhong Du[1,7,8], Andrey A. Ivanov[1,7,8], Deon B Doxie[3], Kavita M Dhodapkar[8,9], Jennifer Carlisle[3,8], Taofeek Owonikoko[3,8], Gabriel Sica[8,10], Yuan Liu[8,11], Suresh Ramalingam[3,8], Madhav V Dhodapkar[3,8], Wei Zhou[3,8*], Xiulei Mo[1,8*], Haian Fu[1,3,7,8*]

[1] Department of Pharmacology and Chemical Biology, Emory University School of Medicine, Atlanta, GA 30322, USA

[2] Department of Obstetrics and Gynecology, The Third Xiangya Hospital, Central South University, Changsha, Hunan 410013, P.R.China

[3] Department of Hematology and Medical Oncology, Emory University, Atlanta, GA 30322, USA

[4] Department of Dermatology, Xiangya Hospital, Central South University, Changsha, 410008, China

[5] The First Affiliated Hospital, Medical School of Xi'an Jiaotong University, Xi'an, Shannxi 710061, P.R.China

[6] Cancer Institute, the Second Affiliated Hospital, Zhejiang University School of Medicine, Hangzhou, Zhejiang 310052, P.R.China

[7] Emory Chemical Biology Discovery Center, Emory University School of Medicine,

Atlanta, GA 30322, USA

[8] Winship Cancer Institute of Emory University, Atlanta, GA 30322, USA.

[9] Aflac Cancer and Blood Disorders Center, Children's Healthcare of Atlanta, Emory University, Atlanta, GA, USA.

[10] Department of Pathology and Laboratory Medicine, Emory University School of Medicine, Atlanta, GA 30322, USA.

[11] Rollins School of Public Health, Emory University, Atlanta, GA, USA.

*Correspondence: Haian Fu, hfu@emory.edu; Xiulei Mo, xmo@emory.edu; Wei Zhou, wzhou2@emory.edu

Lead contact: Haian Fu, hfu@emory.edu

Running title: Genomic mutation-associated immune resistance

Supplementary Figure S1

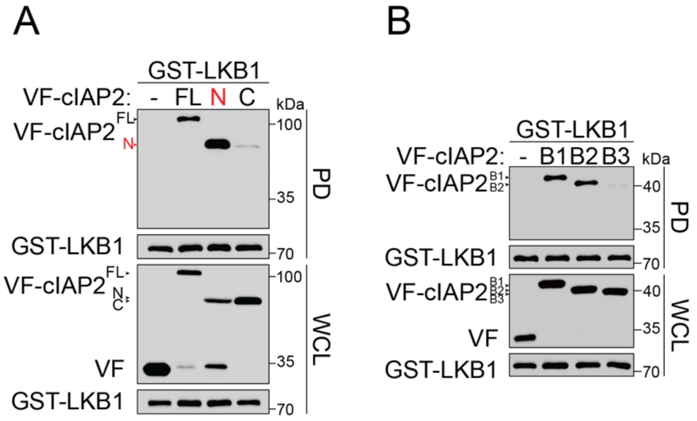

Figure S1. Characterization of LKB1-cIAP2 and JAK1-cIAP2 PPIs, related to Fig. 1. **(A-B)** Immunoblot showing mapping of LKB1-binding domain on cIAP2. Cell lysate from HEK293T cells co-expressing GST-LKB1 and VF-tagged cIAP2 full-length (FL), N-terminal truncation (N), C-terminal truncation (C) or BIR domain truncations (B1, B2 and B3) were subjected to the GST-pulldown as indicated. The pulldown complex and whole cell lysate (WCL) were analyzed by SDS-PAGE and western blot with antibodies as indicated. Source data are provided as a Source Data file. Representative blot of n = 3 independent experiments.

Supplementary Figure S2

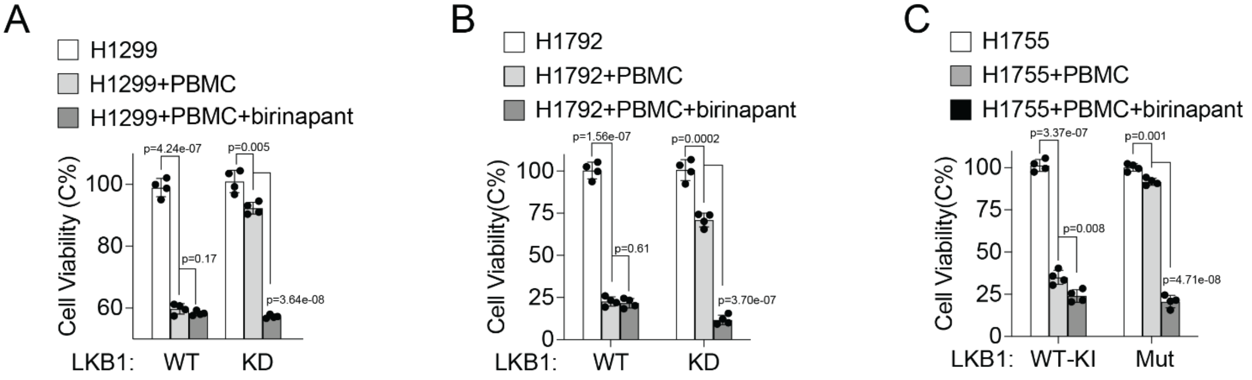

Figure S2. Birinapant-induced sensitization of immune responsiveness in isogenic LKB1-mut cells, related to Fig. 2. **(A)** Bar graph showing cell viability in isogenic LKB1-KD H1299 cells. Parental H1299 (WT) or isogenic LKB1-KD cells were cultured alone or co-cultured with PBMC (E:T=15:1) in the absence or presence of birinapant (150 nM) for 4 days. **(B)** Bar graph showing cell viability in isogenic LKB1- KD H1792 cells. Parental H1792 (WT) or isogenic LKB1-KD cells were cultured alone or co-cultured with PBMC (E:T=10:1) in the absence or presence of birinapant (300 nM) for 4 days. **(C)** Bar graph showing cell viability in isogenic LKB1-WT knockin (WT-KI) H1755 cells. Parental H1755 (LKB-Mut) or isogenic LKB1-WT-KI cells were cultured alone or co-cultured with PBMC (E:T=1:1) in the absence or presence of birinapant (100 nM) for 4 days. Source data are provided as a Source Data file. The data are presented as mean values ± SD from n = 4 independent experiments. P values were calculated by unpaired Student's t-test with two-tailed analysis without adjustments.

Supplementary Figure S3

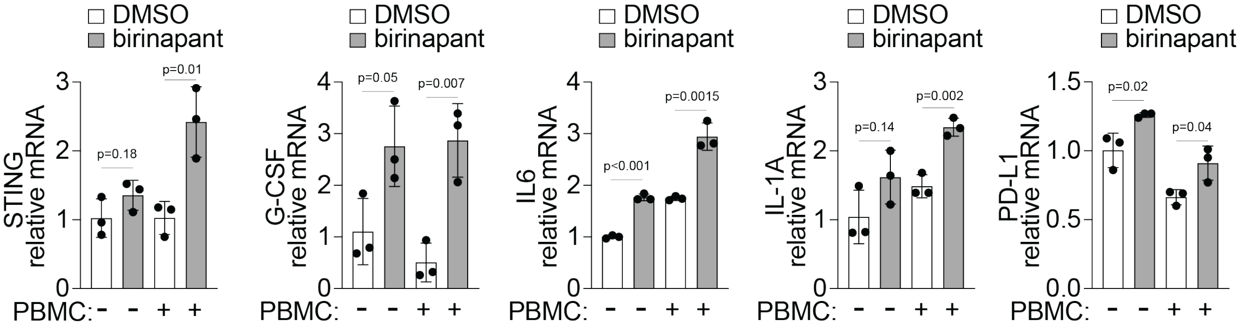

Figure S3. Birinapant-induced immune-dependent STING expression in LKB1-mut cells, related to Fig. 2. Bar graph showing STING, G-CSF, IL6, IL-1A and PD-L1 relative mRNA expression in H1755 cancer cells that were cultured alone or co-culture with immune cells (E:T=1:1). The change of relative mRNA expression of the selected genes was expressed as fold-of-change upon birinapant (50 nM) treatment over DMSO control. Source data are provided as a Source Data file. The data are presented as mean±SD from n = 3 independent experiments. P values were calculated by unpaired Student's t-test with two-tailed analysis without adjustments.

Supplementary Figure S4

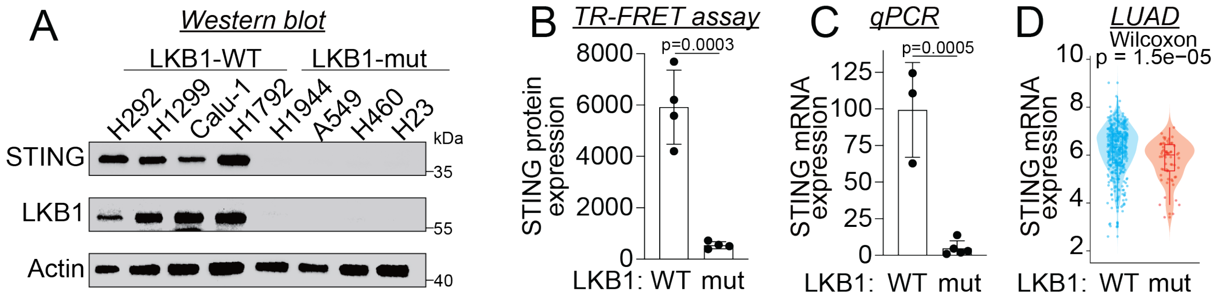

Figure S4. Downregulation of STING expression in LKB1-mut cells, related to Fig. 2. **(A)** Western blot and **(B)** TR-FRET assay analysis of STING protein expression in LUAD cell lines with LKB1-WT (Calu-1, H1299, H1792 and H292) or LKB1-mut (A549, H1944, H23 and H460). **(C)** qPCR assay and **(D)** TCGA analysis of STING mRNA level in LUAD cell lines and patient samples. Source data are provided as a Source Data file. For A, data are presented as one representative blot of n = 3 independent experiments. For B-C, the data are presented as mean±SD of n = 3 independent experiments and P values were calculated by unpaired Student's t-test with two-tailed analysis without adjustments. For D, data was acquired from TCGA-LUAD patient samples (n=511) using TIMER online database and P values was calculated by Wilcoxon test(http://timer.cistrome.org/).

Supplementary Figure S5

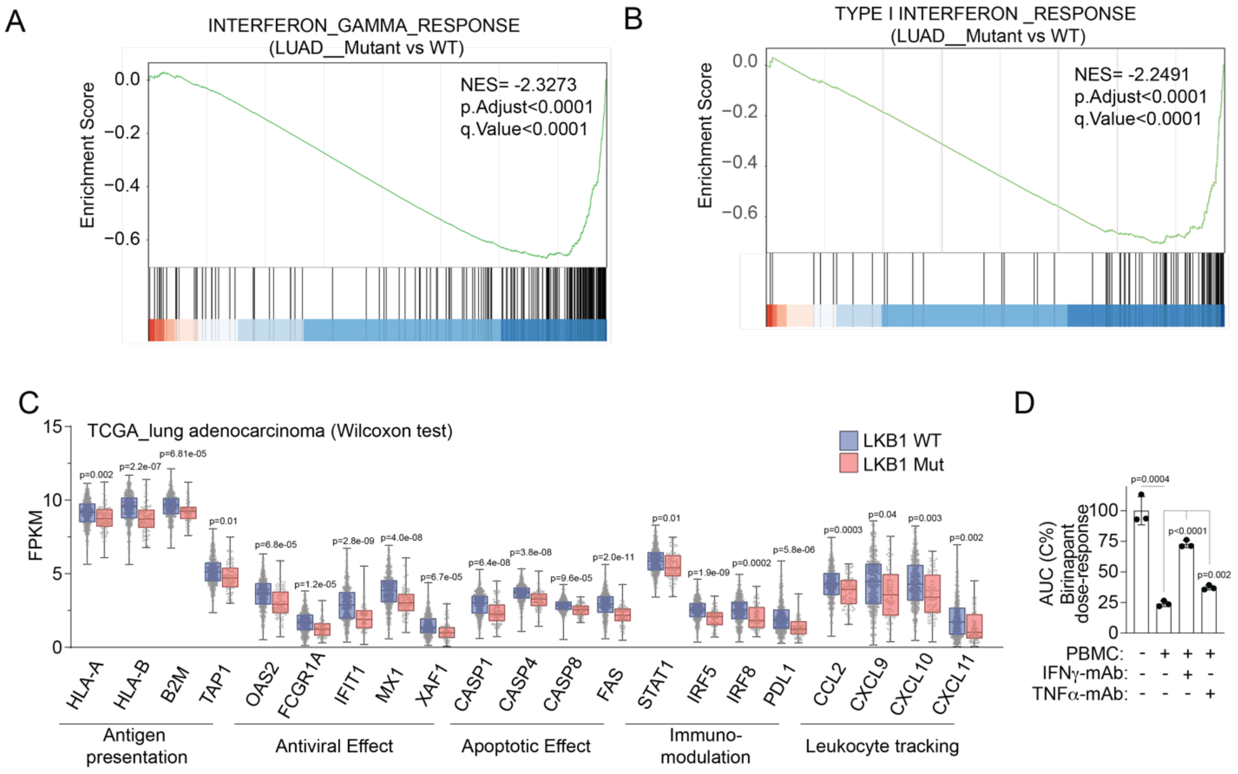

Figure S5. Downregulation of type I interferon and IFNγ pathway in LKB1-mut cells, related to Fig. 3. **(A-B)** Gene set enrichment analysis showing significantly enrichment of differential expression genes from LKB1-mut as compared to LKB1-WT LUAD TCGA samples. **(C)** Downregulation of type I interferon and IFNγ pathway-associated gene in LKB1-mut LUAD TCGA samples. **(D)** Bar graph showing birinapant-induced immune-dependent anti-tumor activity in the presence or absence of IFNγ or TNFα neutralization monoclonal antibodies (mAb). The birinapant-induced immune-dependent anti-tumor activity are presented as mean±SD of the area under the curve (AUC) of the birinapant dose-response curve in PBMC and H1755 cell co-culture assay (n = 3 independent experiments). Source data are provided as a Source Data file. For C and D, P values were calculated by unpaired Student’s t-test with two-tailed analysis without adjustments.

Supplementary Figure S6

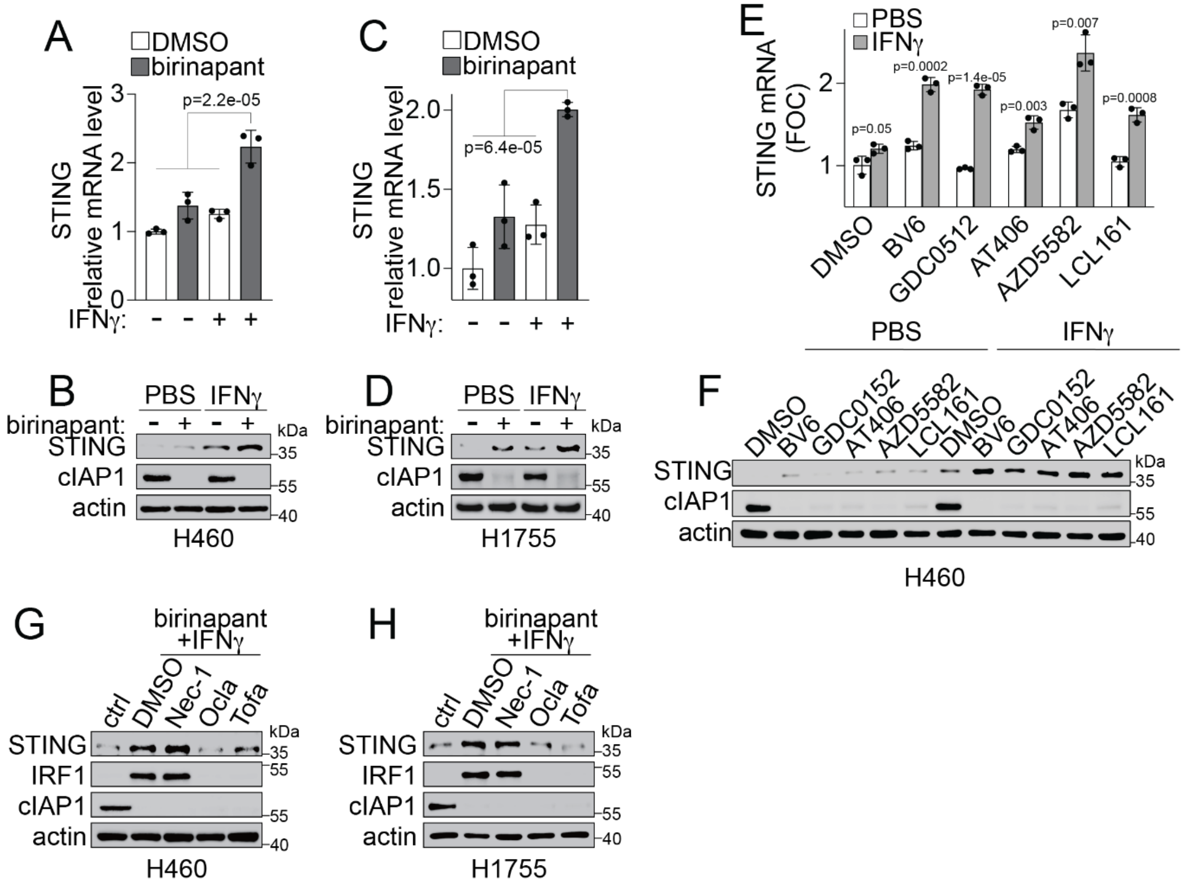

Figure S6. IAP inhibitor-induced IFNγ-dependent STING expression in LKB1-mut cells, related to Fig. 3. **(A-F)** qPCR showing the STING mRNA (A, C and E) and immunoblot showing the in indicated proteins (B, D and F) expression in LKB1-mut cells upon treatment of IAP inhibitor, IFNγ, or in combination. H460 (A-B) and H1755 (C-D) cells were treated with birinapant (500 nM) or other IAP inhibitors (500 nM), IFNγ (5 ng/mL), or in combination as indicated for 24 hours. **(G-H)** Representative immunoblot showing STING protein expression in LKB1-mut cells upon birinapant and IFNγ combination treatment with additional JAK inhibitors. H460 (G) and H1755 (H) cells were treated with birinapant (500 nM) plus IFNg (5 ng/mL) in combination with JAK inhibitors, oclacitinib (Ocla, 10 μM) and tofacitinib (Tofa, 10 μM), or RIPK inhibitor, necrostatin-1

(Nec-1, 10 μM), as indicated. Source data are provided as a Source Data file. For A, C and E, the data are presented as mean±SD from n=3 independent experiments and P values were calculated by unpaired Student's t-test with two-tailed analysis without adjustments. For B, D, F and G-H, data are presented as one representative blot of n = 3 independent experiments.

Supplementary Figure S7

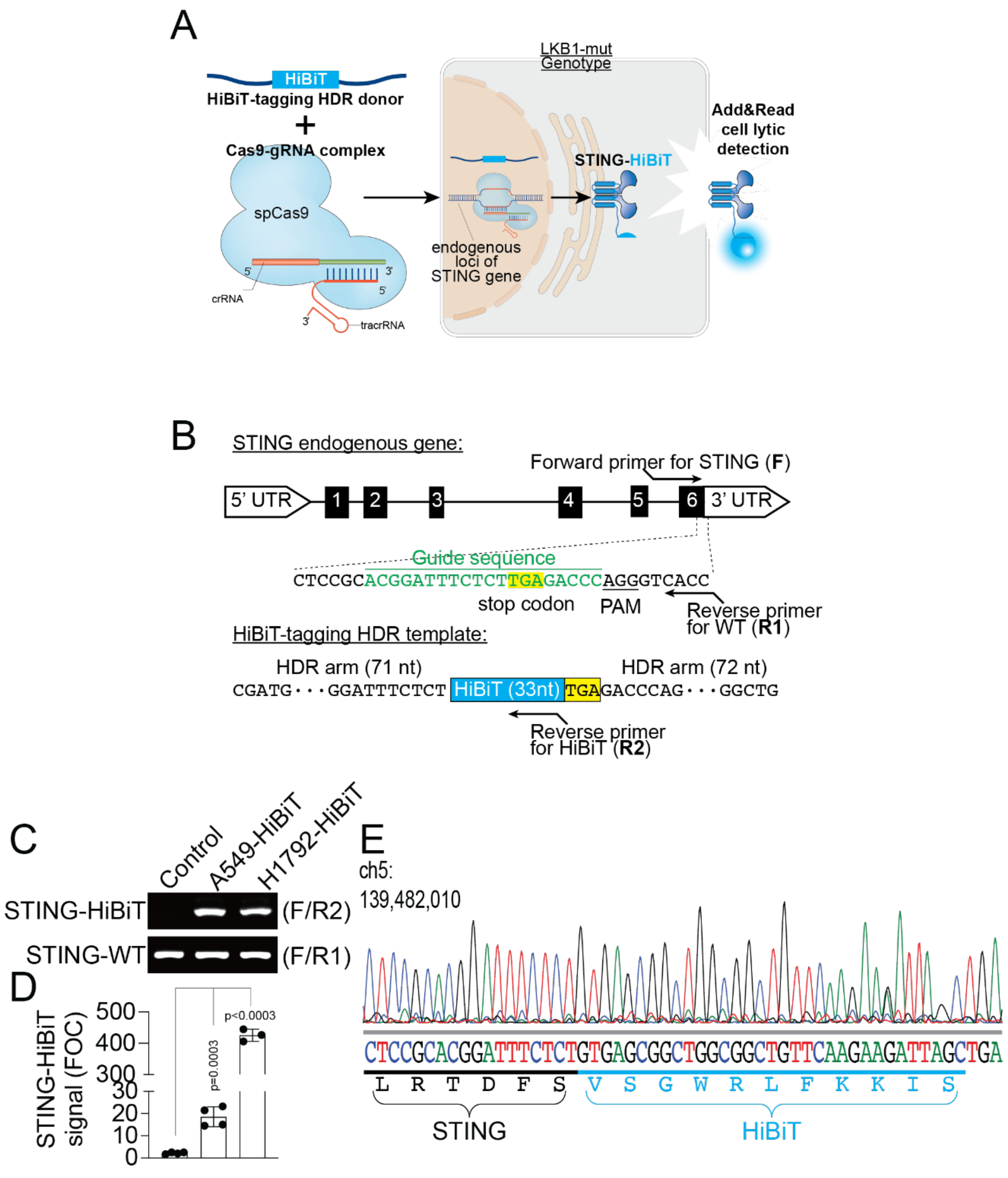

Figure S7. Design and development of STING-HiBiT assay to monitor endogenous STING expression, related to Fig. 3. **(A)** Schematic illustration of CRISPR/Cas9-HiBiT tagging technology for STING-HiBiT cell engineering. **(B)** Schematic illustration of gRNA

and HDR template design. **(C-E)** Validation of STING-HiBiT engineered cells by PCR using primer pair as indicated (C), HiBiT luminescence (D) and sanger sequencing (E). The data in (D) are presented as mean±SD of three independent experiments.

Source data are provided as a Source Data file. For D, the data are presented as mean±SD and P values were calculated by unpaired Student's t-test with two-tailed analysis without adjustments.

Supplementary Figure S8

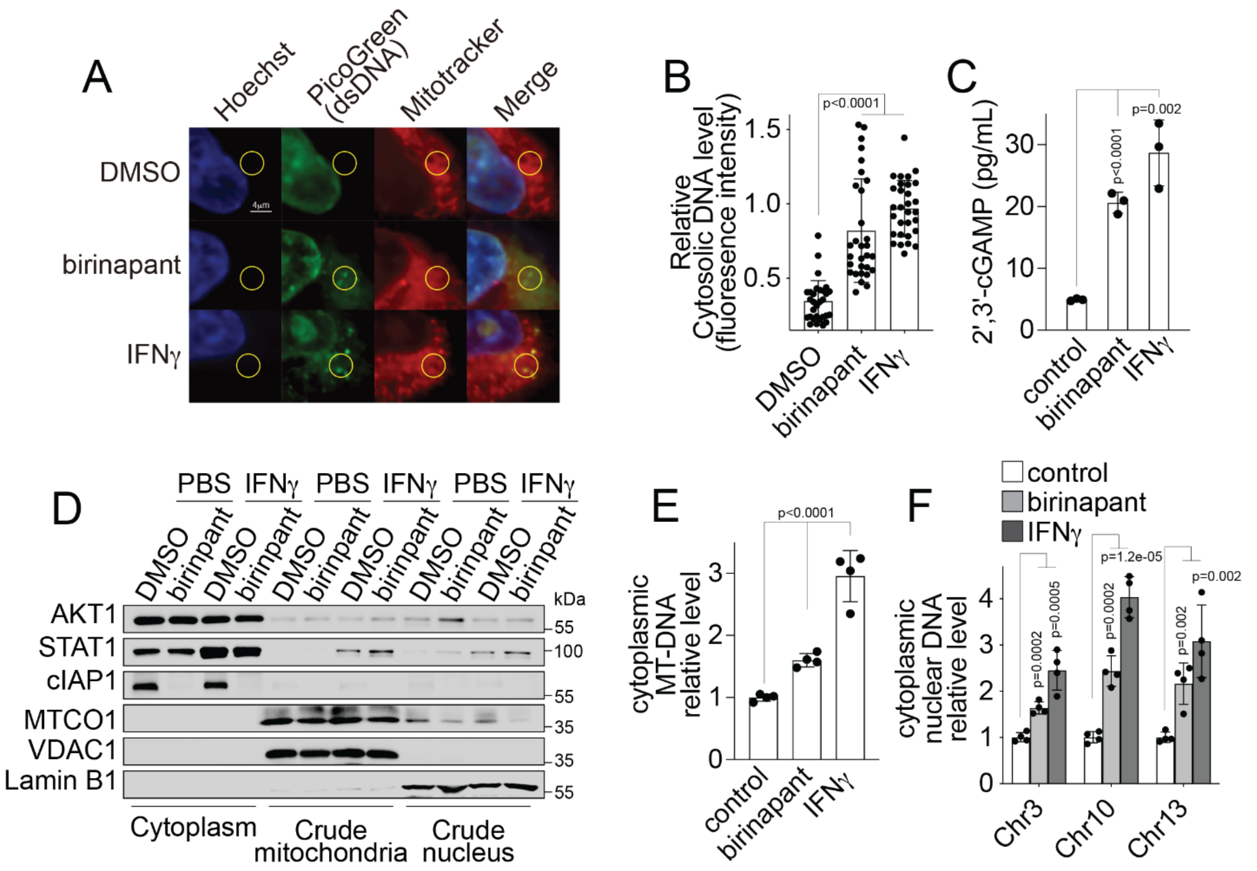

Figure S8. Birinapant- and IFNγ-induced increase of cytosolic DNA in LKB1-mut cells, related to Fig. 4. **(A)** Representative images showing cytosolic DNA staining in A549 cells treated with birinapant (500 nM) or IFNγ (1 ng/mL) for 24 hours and stained with flourescent dyes as indicated. Scale bar: 4 μm. **(B)** Bar graph showing cytosolic DNA quantification from immunostaining. Relative fluorescence intensity was caculated as the ratio of PicoGreen intensity to Mitotracker intersity of n=30 area of interest from n=3 independent experiments. **(C)** Bar grpah showing cGAMP level in A549 cells were treated with birinapant (500 nM) or IFNγ (1 ng/mL) for 24 hours. 2',3'-cGAMP level was presented as mean±SD of n=3 independent experiments. **(D)** Representative immunoblot showing the successful preparation of lysate from cytoplasm. A549 cells were treated with

birinapant (500 nM) or IFNγ (1 ng/mL) for 24 hours and subjected to cell lysate preparation from cytoplasm, mitochondria, and nucleus. Cell lysate was confirmed using representative subcellular compartment resident proteins, such as AKT1 in cytoplasm, VDAC1 and MTCO1 in mitochondria, and Lamin B1 in nucleus. one representative blot of n = 3 independent experiments. **(E-F)** Bar graphs showing birinapant- and IFNγ-induced increase of cytoplasmic DNA released from mitochondria (E, MT-DNA) or nucleus (F, nuclear DNA). Cytoplasmic DNA was quantified by qPCR using MT-DNA or nuclear-DNA specific primers (n = 4 independent experiments). Source data are provided as a Source Data file. For B-C and E-F, the data are presented as mean±SD and P values were calculated by unpaired Student's t-test with two-tailed analysis without adjustments.

Supplementary Figure S9

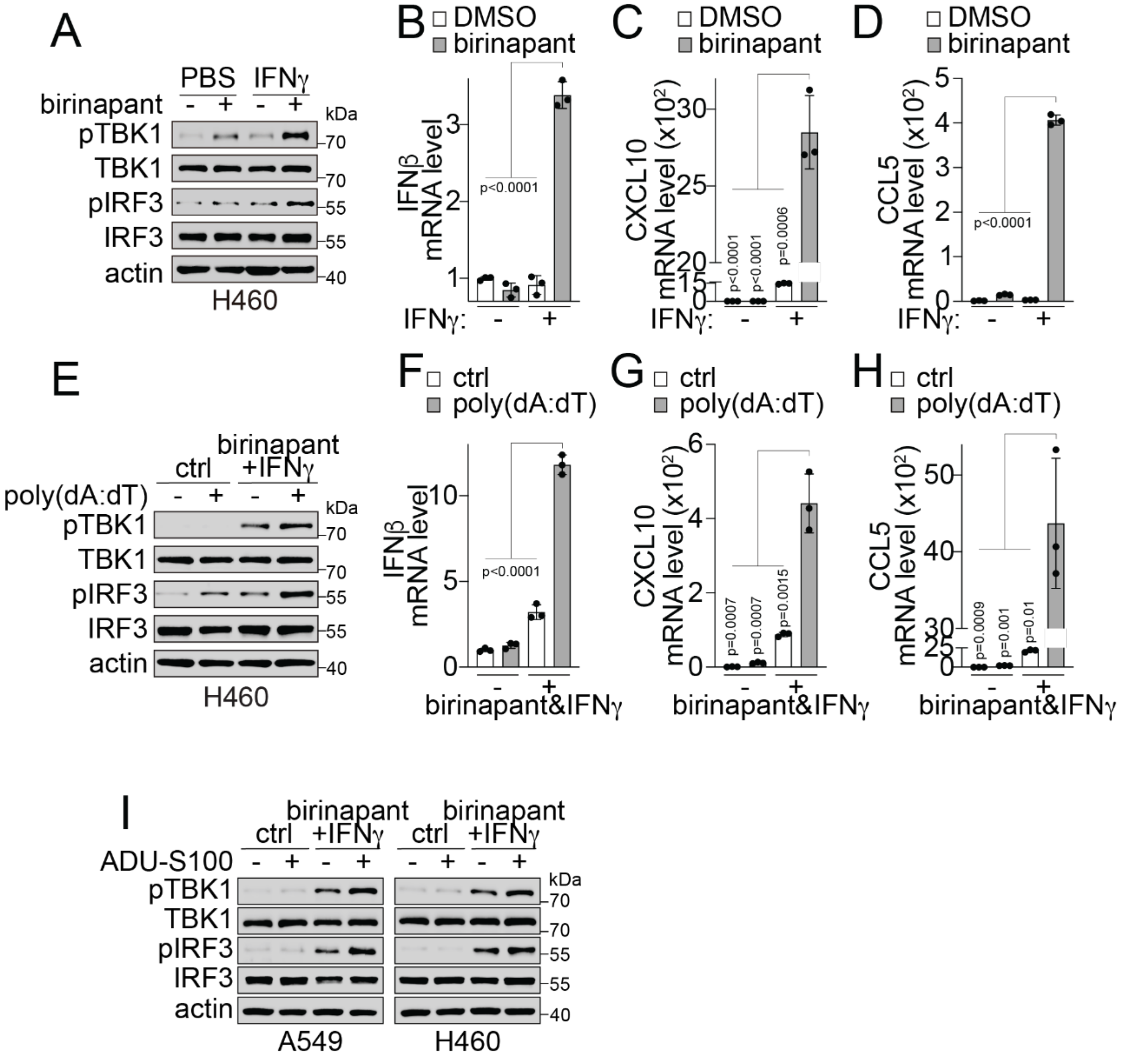

Figure S9. Birinapant synergizes with IFNγ to induce STING-mediated DNA sensing pathway activation in LKB1-mut cells, related to Fig. 4. **(A)** Immunoblot showing indicated proteins in H460 cells treated with birinapant (500 nM) and/or IFNγ (5 ng/mL) for 24 hours as indicated. **(B-D)** qPCR showing mRNA expression level of IFNβ (B), CXCL10 (C) and CCL5 (D) in H460 cells treated with birinapant (500 nM) and/or IFNγ (5 ng/mL) for 24 hours as indicated. **(E)** Immunoblot showing indicated proteins in H460 cells treated with poly(dA:dT) (1 μg/mL) for 4 hours in the presence or absence of 24-hour pre-

treatment with birinapant (500 nM) and IFNγ (5 ng/mL) combination as indicated. **(F-H)** qPCR showing mRNA expression level of IFNβ (F), CXCL10 (G) and CCL5 (H) in H460 cells treated with poly(dA:dT) (1 μg/mL) for 4 hours in the presence or absence of 24-hour pre-treatment with birinapant (500 nM) and IFNγ (5 ng/mL) combination as indicated. **(I)** Immunoblot showing indicated proteins in A549 or H460 cells treated with ADU-S100 (10 μM) for 3 hours in the presence of 24-hour pre-treatment of birinapant (500 nM) plus IFNγ (1 and 5 ng/mL for A549 and H460 cells, respectively). Source data are provided as a Source Data file. For A, E and I, data are presented as one representative blot of n = 3 independent experiments. For B-D and F-H, the data are presented as mean±SD from n = 3 independent experiments and P values were calculated by unpaired Student's t-test with two-tailed analysis without adjustments.

Supplementary Figure S10

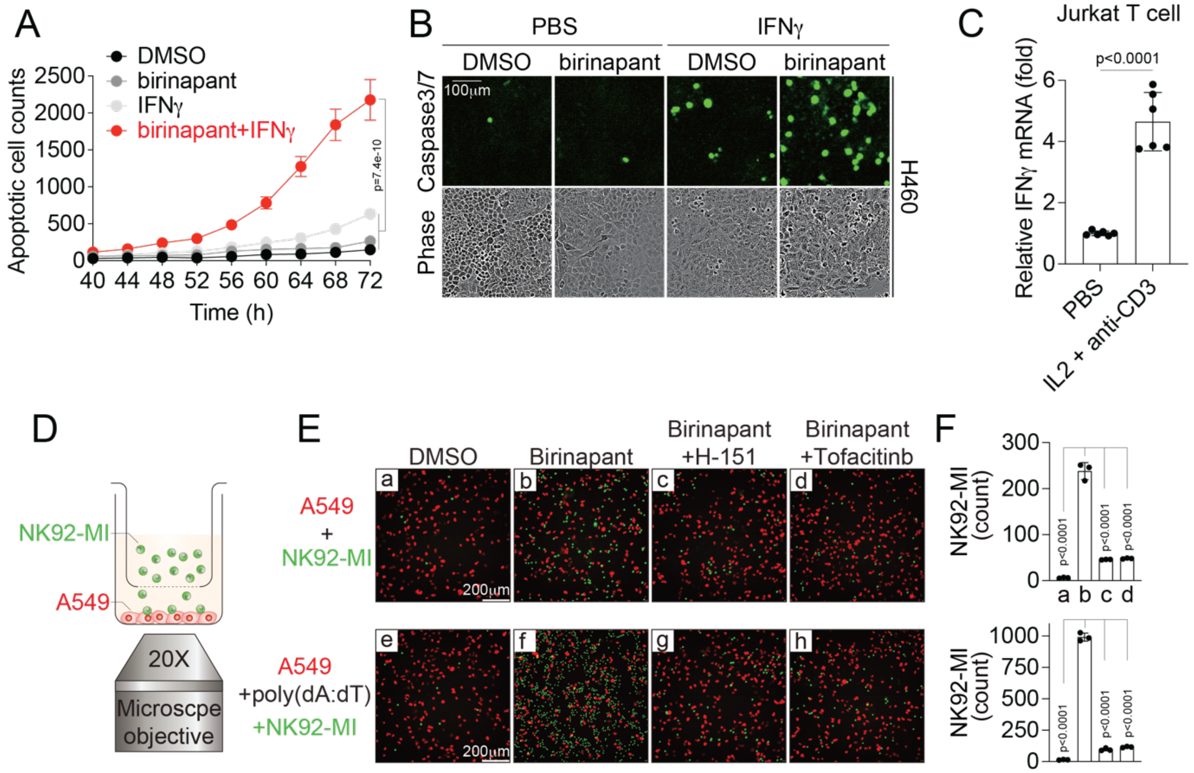

Figure S10. Birinapant induces STING-mediated apoptosis of LKB1-mut cancer cells and migration of immune cells *in vitro*, related to Fig. 5. **(A)** Time-dependent curve of apoptotic cell counts of H460 cells treated with birinapant (500 nM), IFNγ (5 ng/mL) or in combination as indicated. Cell apoptosis was measured and monitored in real-time using DEVD-based fluorogenic caspase-3/7 apoptosis reporter assay up to 72 hours. n = 3 independent experiments. **(B)** Representative images showing birinapant-induced cell apoptosis of H460 cells treated with birinapant (500 nM), IFNγ (5 ng/mL) or in combination as indicated. Green fluorescence (caspase-3/7 apoptosis reporter) and phase-contrast images were acquired using IncuCyte. Scale bar: 100 µm. **(C)** Bar grpah showing activation of IFNγ in Jurkat T cells upon IL2 and anti-CD3 antibody activation in the absence of cancer cells. n = 3 independent experiments. Each dot represents an

individual replicate. **(D)** Schematic illustration of transwell assays for measuring immune cell infiltration *in vitro*. **(E)** Representative fluorescence images showing birinapant-induced NK92-MI cells (IL-2-independent NK-92 cells engineered from transfection with the MFG-hIL2 vector) migration. A549 cells and NK92-MI cells were co-cultured in transwell as shown in (C) and were treated with birinapant (100 nM), poly(dA:dT) (1 μg/mL), or in combination with H151 (5 μM), or JAK inhibitor (JAKi), tofacitinib (10 μM), for 48 hours as indicated. Fluorescence images of A549 cells (red) and infiltrated $CD56^+$ NK92-MI cells (green) were acquired at the endpoint using ImageXpress Micro high-content imaging system. **(F)** Bar graph showing the quantification of infiltrated NK92-MI cells in transwell-based migration assays. n = 3 independent experiments. Source data are provided as a Source Data file. For A, C and F, the data are presented as mean±SD and P values were calculated by unpaired Student's t-test with two-tailed analysis without adjustments.

Supplementary Figure S11

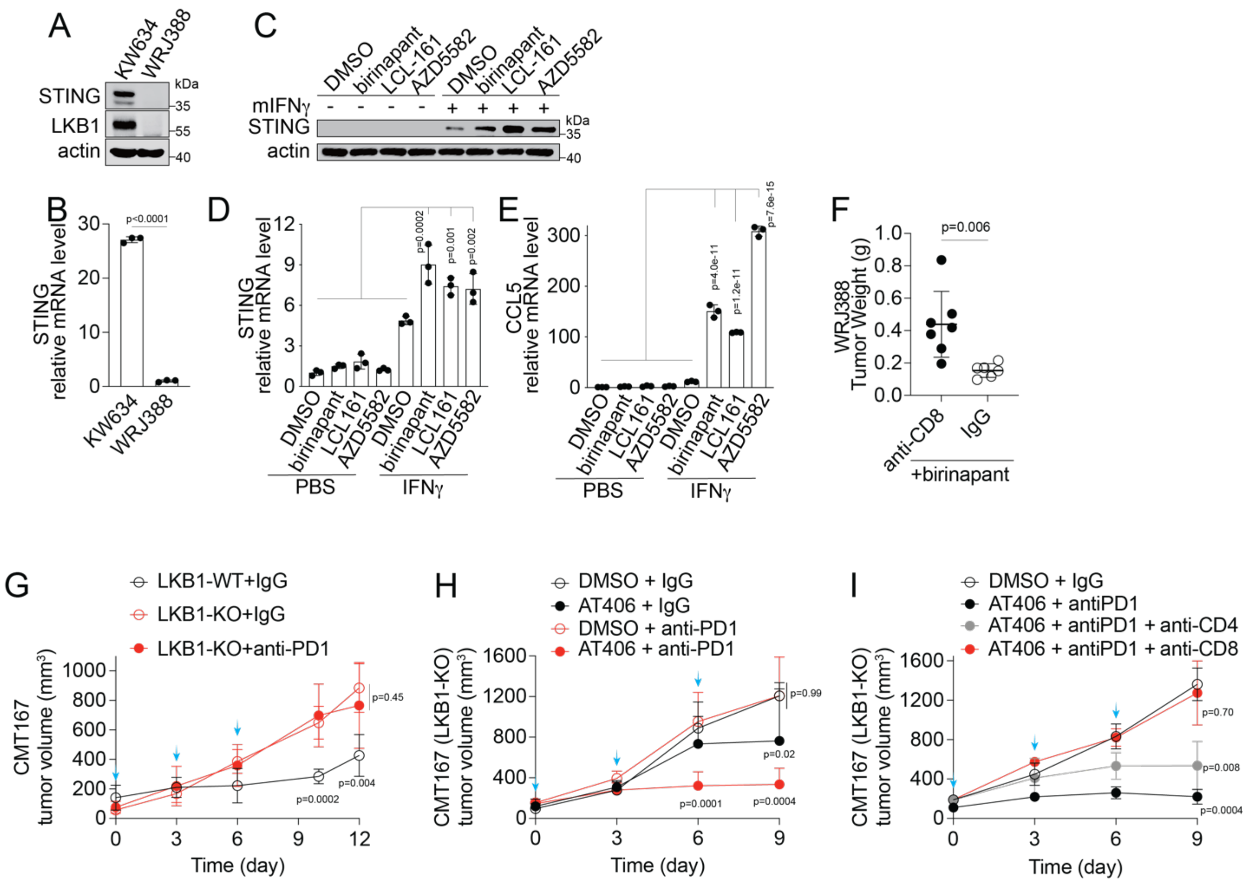

Figure S11. Birinapant synergize with mouse IFNγ to induce STING expression and signaling activation in *Lkb1*-mut mouse lung cancer cells *in vivo*, related to Fig. 6. **(A-B)** STING protein (A) and mRNA (B) expression downregulation in *Lkb1*-mut WRJ388 as compared with *Lkb1*-WT KW634 mouse lung cancer cells. qPCR data are presented as mean±SD of 3 independent experiments. **(C-E)** STING protein expression (C) and STING (D) or CCL5 (E) mRNA expression in WRJ388 cells treated with IAP inhibitors (500 nM) and mouse IFNγ (mIFNγ, 10 ng/mL) as indicated for 24 hours. qPCR data are presented as mean±SD of 3 independent experiments. **(F)** Column dot plot showing WRJ388 tumor weight upon birinapant treatment in the presence of anti-CD8 antibody

(n=7 mice) or IgG control (n=6 mice). The data are presented as mean±SD of the entire experimental cohort. **(G)** *In vivo* tumor growth of isogenic LKB1-KO and LKB1-WT CMT167 cells in immune-competent C57BL/6 mice. Tumor volume were measured in immune-competent mice bearing LKB1-KO CMT167 cells (n=5 mice per group) and the WT counterpart (n=5 mice per group) treated with IgG control or anti-PD1 antibody (200 ug/mouse) as indicated. The data are presented as mean±SD of the entire experimental cohort. **(H)** *In vivo* tumor growth of the LKB1-KO CMT167 cells in immune-competent C57BL/6 mice upon IAPi and anti-PD1 treatment. Tumor volume were measured in immune-competent mice bearing LKB1-KO CMT167 cells (n=5 mice per group) treated with DMSO, AT406 (30 mg/kg), IgG control, anti-PD1 antibody (200 ug/mouse), or combinations as indicated. The data are presented as mean±SD of the entire experimental cohort. **(I)** *In vivo* tumor growth of the LKB1-KO CMT167 cells in immune-competent C57BL/6 mice upon T cell depletion. Tumor volume were measured in immune-competent mice bearing LKB1-KO CMT167 cells (n=5 mice per group) treated with AT406 (30 mg/kg) and anti-PD1 antibody (200 ug/mouse) in the presence of anti-CD8 or anti-CD4 antibodies as indicated. The data are presented as mean±SD of the entire experimental cohort. Source data are provided as a Source Data file. P values were calculated by unpaired Student’s t-test with two-tailed analysis without adjustments.

Supplementary Figure S12

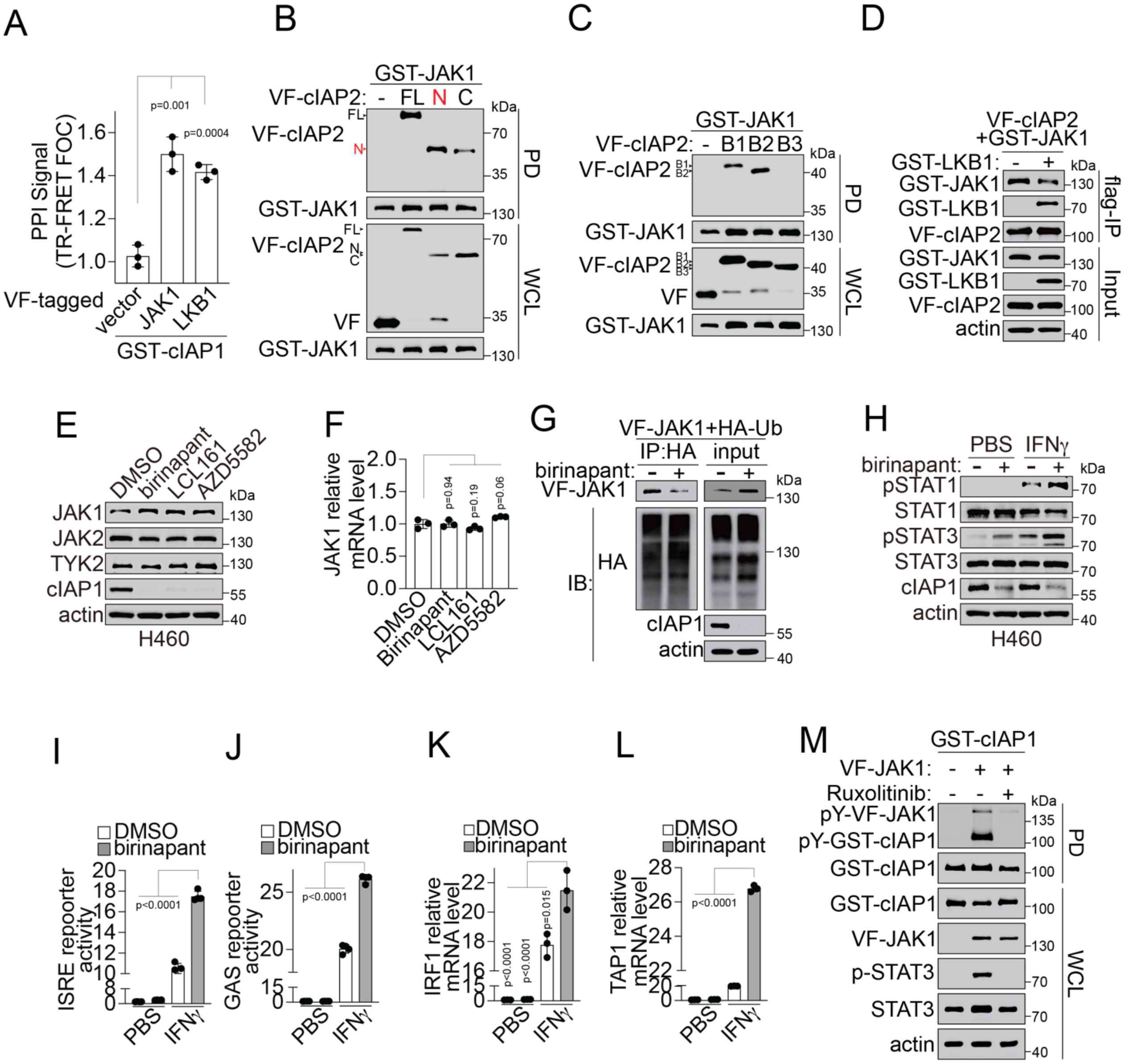

Figure S12. Identification and characterization of LKB1-cIAP1-JAK1 trimolecular interaction, related to Fig. 7. **(A)** TR-FRET PPI signal between cIAP1 with LKB1 and JAK1 using cell lysate from HEK293T cells co-expressing GST-cIAP1 and VF-LKB1 or JAK1 as indicated. n = 3 independent experiments. **(B-C)** Immunoblot showing mapping of JAK1-binding domain on cIAP2. Cell lysate from HEK293T cells co-expressing GST-

JAK1 with and VF-cIAP2 full-length (FL), N-terminal truncation (N), C-terminal truncation (C) or BIR domain truncations (B1, B2, and B3) were subjected to the GST-pulldown as indicated. **(D)** Competitive binding between LKB1 and JAK1 with cIAP2. **(E-F)** JAK1 protein (B) and mRNA (C) expression upon IAP inhibitor treatment in LKB1-mut cells. H460 cells were treated with IAP inhibitors (500 nM) as indicated for 24 hours. mRNA data are presented as mean±SD of 3 independent experiments. **(G)** Birinapant-induced decrease of ubiquitinated JAK1. Cell lysate from HEK293T cells expressing VF-JAK1 and HA-tagged ubiquitin (Ub) with (+) or without (-) 16-hour birinapant (500 nM) plus 6-hour MG132 (20 μM) treatment were subjected to HA-immunoprecipitation. **(H)** Immunoblot showing indicated proteins in H460 cells treated with IFNγ (5 ng/mL) or PBS control for 20 minutes with (+) or without 24-hour pretreatment of birinapant (500 nM). **(I-J)** STAT-driven transcriptional luciferase reporter activity in A549 cells stably expressing ISRE-luc (I) or GAS-luc (J) reporter plasmid treated with birinapant (500 nM), IFNγ (1 ng/mL) or in combination as indicated. The data are presented as as mean±SD from n = 3 independent experiments. **(K-L)** IRF1 (K) and TAP1 (L) mRNA expression in H460 cells treated with birinapant (500 nM), IFNγ (5 ng/mL), or in combination as indicated for 24 hours. The data are presented as mean±SD from n = 3 independent experiments. **(M)** The tyrosine phosphorylated cIAP1 in the co-immunoprecipitated JAK1 complex from cell lysate of HEK293T cells co-expressing GST-cIAP1 with VF-tagged JAK1 were subjected to the GST-pulldown as indicated. Source data are provided as a Source Data file. For B-E, G-H and M, one representative experiment of n = 3 independent experiments. For A, F and I-L, P values were calculated by unpaired Student's t-test with two-tailed analysis without adjustments.
