# Supplementary material for: Uncovering the rewired IAP-JAK regulatory axis as an immune-dependent vulnerability of LKB1-mutant lung cancer
Source: Nat Commun. 2025 Mar 8;16:2324. doi: 10.1038/s41467-025-57297-5 (PMC11890758; doi:10.1038/s41467-025-57297-5)
Supplement: Supplementary file 2 — Description of Additional Supplementary Files [file 41467_2025_57297_MOESM2_ESM.pdf]

Description of Additional Supplementary Files

Supplementary Data 1: detailed oligonucleotide information

Supplementary Data 2: EEBL screen dataset

Supplementary Data 3: DEG list from RNA-seq

Supplementary Data 4: CyTOF panel

Supplementary Data 5: Key Source Table
