# Supplementary material for: Uncovering the rewired IAP-JAK regulatory axis as an immune-dependent vulnerability of LKB1-mutant lung cancer
Source: Nat Commun. 2025 Mar 8;16:2324. doi: 10.1038/s41467-025-57297-5 (PMC11890758; doi:10.1038/s41467-025-57297-5)
Supplement: Supplementary file 8 — Reporting Summary [file 41467_2025_57297_MOESM8_ESM.pdf]

nature portfolio

Corresponding author(s): NCOMMS-23-25213A
Last updated by author(s): 11/17/2024

Reporting Summary

Nature Portfolio wishes to improve the reproducibility of the work that we publish. This form provides structure for consistency and transparency in reporting. For further information on Nature Portfolio policies, see our Editorial Policies and the Editorial Policy Checklist.

Statistics

For all statistical analyses, confirm that the following items are present in the figure legend, table legend, main text, or Methods section.

n/a	Confirmed	
☐	☒	The exact sample size (*n*) for each experimental group/condition, given as a discrete number and unit of measurement
☐	☒	A statement on whether measurements were taken from distinct samples or whether the same sample was measured repeatedly
☐	☒	The statistical test(s) used AND whether they are one- or two-sided<br>*Only common tests should be described solely by name; describe more complex techniques in the Methods section.*
☒	☐	A description of all covariates tested
☒	☐	A description of any assumptions or corrections, such as tests of normality and adjustment for multiple comparisons
☒	☐	A full description of the statistical parameters including central tendency (e.g. means) or other basic estimates (e.g. regression coefficient) AND variation (e.g. standard deviation) or associated estimates of uncertainty (e.g. confidence intervals)
☐	☒	For null hypothesis testing, the test statistic (e.g. *F*, *t*, *r*) with confidence intervals, effect sizes, degrees of freedom and *P* value noted<br>*Give P values as exact values whenever suitable.*
☒	☐	For Bayesian analysis, information on the choice of priors and Markov chain Monte Carlo settings
☒	☐	For hierarchical and complex designs, identification of the appropriate level for tests and full reporting of outcomes
☒	☐	Estimates of effect sizes (e.g. Cohen's *d*, Pearson's *r*), indicating how they were calculated

Our web collection on statistics for biologists contains articles on many of the points above.

Software and code

Policy information about availability of computer code

Data collection	Cell images were acquired using IncuCyte (Sartorius); Cell viability and TR-FRET were collected using PHERAstar FSX (BMG); STING-HiBiT and luciferase reporter assay data was collected using Envision plate reader (PerkinElmer); Transcriptome data were collected using Novogene bulk RNA-seq service; western blot images were collected using ChemiDoc Touch (BioRad)
Data analysis	Statistic analysis: GraphPad Prism v7.0<br>Images processing and analysis: ImageLab (BioRad) and ImageJ (NIH)<br>RNA-seq data analysis: RNAseq data were analyzed using open-source packages from Bioconductor: DeSeq2 for differential gene analysis, and GSEA for gene set enrichment analysis.

For manuscripts utilizing custom algorithms or software that are central to the research but not yet described in published literature, software must be made available to editors and reviewers. We strongly encourage code deposition in a community repository (e.g. GitHub). See the Nature Portfolio guidelines for submitting code & software for further information.

Data

Policy information about availability of data

All manuscripts must include a data availability statement. This statement should provide the following information, where applicable:

- Accession codes, unique identifiers, or web links for publicly available datasets
- A description of any restrictions on data availability
- For clinical datasets or third party data, please ensure that the statement adheres to our policy

Analyzed small molecule screening data sets and mRNA sequencing data sets will be available through CTD2 data portal (https://ocg.cancer.gov/programs/ctd2/data-portal).

Research involving human participants, their data, or biological material

Policy information about studies with human participants or human data. See also policy information about sex, gender (identity/presentation), and sexual orientation and race, ethnicity and racism.

Reporting on sex and gender	NA
Reporting on race, ethnicity, or other socially relevant groupings	NA
Population characteristics	NA
Recruitment	NA
Ethics oversight	NA

Note that full information on the approval of the study protocol must also be provided in the manuscript.

Field-specific reporting

Please select the one below that is the best fit for your research. If you are not sure, read the appropriate sections before making your selection.

☒ Life sciences ☐ Behavioural & social sciences ☐ Ecological, evolutionary & environmental sciences

For a reference copy of the document with all sections, see nature.com/documents/nr-reporting-summary-flat.pdf

Life sciences study design

All studies must disclose on these points even when the disclosure is negative.

Sample size	For in vitro cell line or biochemical studies, three independent experiments were selected to ensure statistical power;<br>For in vivo animal studies, no statistical methods were used to predetermine the sample size. Sample sizes were chosen based on prior experience and pilot studies for detecting statistically significant differences between samples. In compliance to IACUC guidelines, a minimal number of animals for a statistically significant results was used.
Data exclusions	No data was excluded from the studies
Replication	All attempts at replication were successful. Biological and technical replicates of each experiment is stated in the figure legend and all attempts were successful. Furthermore, key findings were validated independently by multiple personnel in different model systems.
Randomization	For in vivo animal studies, mice were randomly assigned to different treatment groups.
Blinding	Analysis of chemical screen and RNA-seq analysis were blindly conducted using softwares and open source scripts. Investigators were not blinded to conduct other in vitro and in vivo studies, as knowledges of this information was essential to conduct the studies.

Reporting for specific materials, systems and methods

We require information from authors about some types of materials, experimental systems and methods used in many studies. Here, indicate whether each material, system or method listed is relevant to your study. If you are not sure if a list item applies to your research, read the appropriate section before selecting a response.

Materials & experimental systems

n/a	Involved in the study
	☒ Antibodies
	☒ Eukaryotic cell lines
☒	Palaeontology and archaeology
	☒ Animals and other organisms
☒	Clinical data
☒	Dual use research of concern
☒	Plants

Methods

n/a	Involved in the study
☒	ChIP-seq
☒	Flow cytometry
☒	MRI-based neuroimaging

Antibodies

Antibodies used

CD3 monoclonal antibody (OKT3) Thermo Fisher Scientific Cat# 16-0037-81, RRID:AB_468854
DYKDDDDK Tag (anti-Flag) Rabbit mAb Cell Signaling Technology Cat# 14793, RRID:AB_2572291
Tb cryptate-conjugated anti-GST antibody Cisbio Bioassays Cat# 61GSTTLB
Mouse monoclonal anti-GST (B-14) antibody Santa Cruz Biotechnology Cat# sc-138, RRID:AB_627677
Rabbit Anti-HA-Tag Monoclonal Antibody Cell Signaling Technology Cat# 3724, RRID:AB_1549585
Anti-β-Actin Antibody Sigma-Aldrich Cat# A5441, RRID:AB_476744
STING (D2P2F) antibody Cell Signaling Technology Cat# 13647, RRID:AB_2732796
TBK1/NAK (D1B4) Rabbit mAb Cell Signaling Technology Cat# 3504, RRID:AB_2255663
Phospho-TBK1/NAK (Ser172) (D52C2) XP Rabbit monoclonal Antibody Cell Signaling Technology Cat# 5483, RRID:AB_10693472
IRF3 antibody Abcam Cat# ab68481, RRID:AB_11155653
IRF3 (phospho S386) antibody Abcam Cat# ab76493, RRID:AB_1523836
LKB1 Monoclonal Antibody Thermo Fisher Scientific Cat# AHO1392, RRID:AB_2536337
Jak1 (D1T6W) Mouse mAb antibody Cell Signaling Technology Cat# 50996, RRID:AB_2716281
Jak2 (D2E12) XP Rabbit mAb antibody Cell Signaling Technology Cat# 3230, RRID:AB_2128522
Tyk2 (D4I5T) Rabbit mAb antibody Cell Signaling Technology Cat# 14193, RRID:AB_2798419
Stat1 Antibody Cell Signaling Technology Cat# 9172, RRID:AB_2198300
Phospho-Stat1 (Tyr701) (D4A7) Rabbit mAb Cell Signaling Technology Cat# 7649, RRID:AB_10950970
Stat3 (79D7) Rabbit mAb antibody Cell Signaling Technology Cat# 4904, RRID:AB_331269
Phospho-Stat3 (Tyr705) (D3A7) XP Rabbit mAb Cell Signaling Technology Cat# 9145, RRID:AB_2491009
c-IAP1 (D5G9) Rabbit mAb Cell Signaling Technology Cat# 7065, RRID:AB_10890862
Anti-Ubiquitin Antibody Cell Signaling Technology Cat# 3933, RRID:AB_2180538
Peroxidase-AffiniPure Goat Anti-Mouse IgG (H + L) antibody Jackson ImmunoResearch Labs Cat# 115-035-003, RRID:AB_10015289
Peroxidase-AffiniPure Goat Anti-Rabbit IgG (H+L) antibody Jackson ImmunoResearch Labs Cat# 111-035-003, RRID:AB_2313567
Mouse CD8 antibody (IHC) Abcam Cat# ab237723

Validation

Validation statement for each antibody is provided on the manufacturer's website.

Eukaryotic cell lines

Policy information about cell lines and Sex and Gender in Research

Cell line source(s)

Peripheral Blood Mononuclear Cells, Human, Normal ATCC Cat# PCS-800-011
HEK293T ATCC Cat# CRL-3216; RRID: CVCL_0063
A549 ATCC Cat# CCL-185, RRID:CVCL_0023
H1792 ATCC Cat# CRL-5895, RRID:CVCL_1495
H1299 ATCC Cat# CRL-5803, RRID:CVCL_0060
H292 ATCC Cat# CRL-1848, RRID:CVCL_0455
Calu-1 ATCC Cat# HTB-54, RRID:CVCL_0608
H1944 ATCC Cat# CRL-5907, RRID:CVCL_1508
H23 ATCC Cat# CRL-5800, RRID:CVCL_1547
H460 ATCC Cat# HTB-177, RRID:CVCL_0459
H1755 ATCC Cat# CRL-5892, RRID:CVCL_1492
Incucyte® A549 NucLight Red Cells Sartorius Cat# 4491
Jurkat T cells ATCC Cat# TIB-152
NK92-MI cells ATCC Cat# CRL-2408
Primary normal CD8+ T cells Stemcell Cat# 200-0164
Primary normal CD56+ NK cells Stemcell Cat# 70037
WRJ388 Winship Cancer Institute (Jin et al., 2021)
KW634 Gifted from Dr. Kwok-Kin Wong (Gandhi et al., 2009)

Authentication

All cell lines were authenticated with STR profiling

Mycoplasma contamination

All cell lines were tested negatively for mycoplasma contamination

Commonly misidentified lines (See ICLAC register)	None of the cell lines used are listed as commonly misidentified lines in ICLAC database.

Animals and other research organisms

Policy information about studies involving animals; ARRIVE guidelines recommended for reporting animal research, and Sex and Gender in Research

Laboratory animals	Immune-competent KL mice Emory Winship Cancer Institute (Gilbert-Ross et al., 2017; Jin et al., 2021)<br><br>Immune-deficient nude mice Harlan Laboratories Strains: Hsd: Athymic Nude-Foxn1nu
Wild animals	NA
Reporting on sex	Both female and male were considered during studies
Field-collected samples	The study did not involve samples collected from field
Ethics oversight	All animal experiments were performed in accordance with protocols approved by Emory University IACUC.

Note that full information on the approval of the study protocol must also be provided in the manuscript.
